# Nonadherence to treatment in lung transplant recipients: a matter of life and death

**DOI:** 10.1590/S1806-37132015000100012

**Published:** 2015

**Authors:** André Nathan Costa, Elaine Marques Hojaij, Liliane Saraiva de Mello, Felipe Xavier de Melo, Priscila Cilene Leon Bueno de Camargo, Silvia Vidal Campos, Jose Eduardo Afonso, Rafael Medeiros Carraro, Ricardo Henrique de Oliveira Braga Teixeira

**Affiliations:** University of São Paulo, School of Medicine, Hospital das Clínicas, São Paulo, Brazil, Department of Pulmonology, Instituto do Coração - InCor, Heart Institute - University of São Paulo School of Medicine Hospital das Clínicas, São Paulo, Brazil; University of São Paulo, School of Medicine, Hospital das Clínicas, São Paulo, Brazil, Departments of Psychology and Lung Transplantation, Instituto do Coração - InCor, Heart Institute - University of São Paulo School of Medicine Hospital das Clínicas, São Paulo, Brazil; University of São Paulo, School of Medicine, Hospital das Clínicas, São Paulo, Brazil, Department of Lung Transplantation, Instituto do Coração - InCor, Heart Institute - University of São Paulo School of Medicine Hospital das Clínicas, São Paulo, Brazil; University of São Paulo, School of Medicine, Hospital das Clínicas, São Paulo, Brazil, Department of Pulmonology, Instituto do Coração - InCor, Heart Institute - University of São Paulo School of Medicine Hospital das Clínicas, São Paulo, Brazil; University of São Paulo, School of Medicine, Hospital das Clínicas, São Paulo, Brazil, Department of Pulmonology, Instituto do Coração - InCor, Heart Institute - University of São Paulo School of Medicine Hospital das Clínicas, São Paulo, Brazil; University of São Paulo, School of Medicine, Hospital das Clínicas, São Paulo, Brazil, Instituto do Coração - InCor, Heart Institute - University of São Paulo School of Medicine Hospital das Clínicas, São Paulo, Brazil; University of São Paulo, School of Medicine, Hospital das Clínicas, São Paulo, Brazil, Department of Pulmonology, Instituto do Coração - InCor, Heart Institute - University of São Paulo School of Medicine Hospital das Clínicas, São Paulo, Brazil; University of São Paulo, School of Medicine, Hospital das Clínicas, São Paulo, Brazil, Department of Pulmonology, Instituto do Coração - InCor, Heart Institute - University of São Paulo School of Medicine Hospital das Clínicas, São Paulo, Brazil; University of São Paulo, School of Medicine, Hospital das Clínicas, São Paulo, Brazil, Department of Pulmonology; and Clinical Coordinator, Lung Transplant Group, Instituto do Coração - InCor, Heart Institute - University of São Paulo School of Medicine Hospital das Clínicas, São Paulo, Brazil

To the Editor: 

Lung transplantation is a complex intervention, requiring strict adherence to a very specific medical regimen, which involves not only drug taking but also a fairly restrictive daily routine. The extent to which patients adhere to the prescribed regimen plays a key role in achieving optimal transplantation outcomes.^(^ 1 ^)^ Therefore, adherence to treatment is of great importance in the care of lung transplant recipients. The concept of adherence implies active participation by patients, who must understand their disease and the proposed treatment and strictly follow the recommendations of the health care team.^(^ 1 ^)^ The World Health Organization proposes a close partnership among physicians, multidisciplinary staff, and patients, in order to improve treatment adherence.^(^ 2 ^)^ The recent death of an adolescent female who underwent lung transplantation in the Lung Transplantation Department of the University of São Paulo School of Medicine *Hospital das Clínicas Instituto do Coração* (InCor, Heart Institute), located in the city of São Paulo, Brazil, and who died because of treatment nonadherence raised great concern about this issue, leading us to revise our multidisciplinary approach to patients and review the current knowledge of treatment adherence. 

An 18-year-old female patient underwent double lung transplantation for end-stage cystic fibrosis. Initial immunosuppressive therapy included basiliximab and methylprednisolone, being followed by maintenance treatment with cyclosporine (adjusted to blood levels), mycophenolate, and prednisone. The patient remained stable for a period of one year and five months, after which she presented with acute progressive shortness of breath, hypoxemia, loss of lung function, and diffuse ground-glass opacities on HRCT scans. Although her outpatient prescription drugs included cyclosporine, mycophenolate, prednisone, itraconazole, and trimethoprim-sulfamethoxazole, her cyclosporine blood levels were far below the minimum target. When queried, her caregiver admitted that, despite his efforts, she had not been taking her medication as prescribed in the past month and had been smoking narghile in her spare time. An open lung biopsy revealed grade A3 acute rejection, chronic airway rejection or bronchiolitis obliterans (C1), chronic vascular rejection (D), and organizing pneumonia. She was treated with rabbit antithymocyte globulin and corticosteroids but died as a result of alveolar hemorrhage and multiple infectious complications. 

Recent studies have shown that as many as 25-50% of chronic disease patients can be considered nonadherers, nonadherence being temporary in some and permanent in others.^(^ 1 ^,^ 2 ^)^ Among transplant recipients, nonadherence rates can be as high as 80%, especially in adolescent patients.^(^ 3 ^)^ Although different methods and definitions of nonadherence (e.g., missed medication doses, delayed medication use, and dose modification) can influence the aforementioned rates,^(^ 1 ^,^ 4 ^)^ nonadherence is undoubtedly an issue of great importance. 

For lung transplant recipients, relief from the incapacitating symptoms of chronic lung disease comes at a price: long-term treatment; a complex therapeutic regimen; drug side effects; a restrictive diet; limited alcohol use; smoking cessation; and constraints on peer socialization. After the initial relief of the chronic symptoms, some patients lose the motivation to follow the strict rules required in order to maintain the graft. Therefore, the immediate relief (or alleviation of anxious feelings) can reduce or negate the positive effects of long-term graft duration, in that it can lead to abandonment of the use of the prescribed medication. Special attention should be given to certain populations of lung transplant recipients. Being an adolescent is a predisposing factor for poor adherence to treatment because of the characteristic feeling of invulnerability in such individuals.^(^ 1 ^)^ Most of the patients at our facility are cystic fibrosis patients, who are exactly in this age group and whose psychological profile increases the risk of nonadherence. However, noncompliance with physician recommendations cannot be understood simply as disobedience. Patients should not be coerced, made to feel guilty, or punished, the staff remaining in the comfortable position that it is up to patients to do exactly as they are told. Blaming patients for losing their graft because of nonadherence is not the correct way to deal with the situation. Health professionals should be trained in adherence assessment and management, because communication skills training with a focus on adherence management results in significantly higher adherence rates.^(^ 3 ^,^ 5 ^)^

Environmental factors that affect the extent to which patients will adhere to the recommended treatment should be identified.^(^ 5 ^,^ 6 ^)^ Notable among such factors are patient beliefs regarding their disease and its treatment; transient emotional and cognitive problems; the quality of social support received; and an established relationship with the health care team.^(^ 5 ^)^ Strategies to improve patient adherence to the therapeutic regimen should be implemented on the basis of individual characteristics and needs.^(^ 5 ^)^ Finally, the health care team and patients should form a cohesive group. 

Given that treatment nonadherence is a complex and multidimensional problem, none of the aforementioned efforts will ever produce a one-size-fits-all solution to nonadherence. Nevertheless, transplant teams should routinely revise and improve their multidisciplinary approach to patients. Further research and education are needed in order to gain a better understanding of how patient characteristics affect adherence to treatment. 
